# Composite Hydrogels Based on Cross-Linked Chitosan and Low Molecular Weight Hyaluronic Acid for Tissue Engineering

**DOI:** 10.3390/polym15102371

**Published:** 2023-05-19

**Authors:** Maria Drozdova, Marina Vodyakova, Tatiana Tolstova, Marina Chernogortseva, Nikita Sazhnev, Tatiana Demina, Nadezhda Aksenova, Peter Timashev, Nataliya Kildeeva, Elena Markvicheva

**Affiliations:** 1 Shemyakin & Ovchinnikov Institute of Bioorganic Chemistry, Russian Academy of Sciences, 16/10 Miklukho-Maklaya Str., 117997 Moscow, Russia; 2 Department of Chemistry and Technology of Polymer Materials and Nanocomposites, The Kosygin Russian State University, 1 Malaya Kaluzhskaya Str., 119071 Moscow, Russia; 3 Enikolopov Institute of Synthetic Polymeric Materials, Russian Academy of Sciences, 70 Profsouznaya Str., 117393 Moscow, Russia; 4 World-Class Research Center “Digital Biodesign and Personalized Healthcare”, Sechenov First Moscow State Medical University, 8-2 Trubetskaya Str., 119991 Moscow, Russia; 5 Semenov Federal Research Center for Chemical Physics, Russian Academy of Sciences, 4 Kosygina Str., 119991 Moscow, Russia; 6 Institute for Regenerative Medicine, Sechenov First Moscow State Medical University, 8-2 Trubetskaya Str., 119991 Moscow, Russia; 7 Chemistry Department, Lomonosov Moscow State University, 1-3 Leninskie Gory, 119991 Moscow, Russia

**Keywords:** chitosan, hyaluronic acid, genipin, covalently cross-liked chitosan, L929 mouse fibroblasts, tissue engineering

## Abstract

The objectives of the study were as follows: (1) to develop two methods for the preparation of macroporous composite chitosan/hyaluronic acid (Ch/HA) hydrogels based on covalently cross-linked Ch and low molecular weight (Mw) HA (5 and 30 kDa); (2) to investigate some properties (swelling and in vitro degradation) and structures of the hydrogels; (3) to evaluate the hydrogels in vitro as potential biodegradable matrices for tissue engineering. Chitosan was cross-linked with either genipin (Gen) or glutaraldehyde (GA). Method 1 allowed the distribution of HA macromolecules within the hydrogel (bulk modification). In Method 2, hyaluronic acid formed a polyelectrolyte complex with Ch over the hydrogel surface (surface modification). By varying compositions of the Ch/HA hydrogels, highly porous interconnected structures (with mean pore sizes of 50–450 μm) were fabricated and studied using confocal laser scanning microscopy (CLSM). Mouse fibroblasts (L929) were cultured in the hydrogels for 7 days. Cell growth and proliferation within the hydrogel samples were studied via MTT-assay. The entrapment of low molecular weight HA was found to result in an enhancement of cell growth in the Ch/HA hydrogels compared to that in the Ch matrices. The Ch/HA hydrogels after bulk modification promoted better cell adhesion, growth and proliferation than the samples prepared by using Method 2 (surface modification).

## 1. Introduction

Polysaccharide hydrogels are of great interest for tissue engineering. Their resemblance to living tissues mimics the natural three-dimensional extracellular matrix (ECM) and promotes cell attachment, proliferation and stem cell differentiation [1]. Chitosan is a biocompatible and biodegradable polymer of natural origin with antimicrobial and biologically adhesive properties [2]. Chitosan is widely employed for tissue engineering, as evidenced by an ever-increasing number of publications [3]. For instance, it is applied in the fields of skin [4], bone [5] and cartilage [6] tissue engineering. However, the rather low mechanical strength of covalently non-cross-linked chitosan matrices could lead to low cell adhesion [7]. The matrices should provide appropriate rigidity to promote cell adhesion and characteristic morphology, and, as a result, an increase of cell migration, growth and differentiation.

Mechanical properties could be enhanced by cross-linking hydrogel matrices [8,9]. There are several cross-linking agents commonly used for this purpose. For instance, glutaraldehyde was proposed as a cross-linking agent to stabilize chitosan [8]. Another promising cross-linker, which should be mentioned here, is the natural compound genipin [10].

However, the covalent cross-linking of chitosan could reduce hydrogel swelling and degradation because of a decrease of chitosan free amino and hydroxyl groups involved in the cross-linking reaction [11]. Adjusting the hydrophilic–hydrophobic balance of the matrices could affect cell adhesion and, as a result, could influence cell proliferation and/or differentiation. The creation of composite materials can be one approach to this.

As is well-known, hyaluronan is promising for many biomedical applications [12]. Being a natural polysaccharide, hyaluronic acid is a key component of ECM and occurs in many connective tissues (such as skin and synovial fluid). Hyaluronic acid acts as a joint lubricant supporting tissue regeneration, hydration and elasticity. Hyaluronic acid can promote these processes due to its polyelectrolyte nature, high water-holding capacity and high viscosity as well as gel-forming ability. However, the biological activity of hyaluronic acid depends on its molecular weight [13]. In nature, the molecular weight of HA can vary within a wide range of 10^3^–10^7^ Da [12,13]. Native HA macromolecules (e.g., in a normal synovial fluid) have rather high molecular weights, in particular more than 1 MDa, and HA oligomers (up to 10 kDa) could be also found [14]. Hyaluronic acid with high molecular weight can provide tissue integrity, water homeostasis and possesses immunosuppressive and anti-inflammatory properties [15]. However, the pro-proliferative and differentiation induction properties of HA with high molecular weight are controversial [16,17], whereas HA with low molecular weight (<400 kDa) signals injury and can initiate an inflammatory response [18]. Additionally, HA can stimulate angiogenic activity and enhance cell proliferation [19]. Hyaluronic acid oligosaccharides were shown to exhibit pro-antigenic properties and to stimulate endothelial cell migration and proliferation [20].

Composite Ch/HA matrices can be prepared by using various methods. Because Ch and HA are oppositely charged polyelectrolytes, they can form a water-insoluble polyelectrolyte complex. Sodium hyaluronate/chitosan polyelectrolyte complexes have been used for dental pulp regeneration [21], cartilage repair [22] and the regeneration of periodontal tissues [23]. However, HA/Ch complexes are rather unstable at philological conditions [21,24]. Therefore, to prepare rather stable composite Ch/HA matrices and, as a result, to enhance their mechanical properties, the covalent cross-linking of Ch molecules was proposed. This approach allows enhancement of both the stability and flexibility of the composite Ch/HA matrices [9,25]. It should be mentioned that in these cases, HA of high molecular weight (>600 kDa) was used.

In the current study, for the first time, HA with low molecular weight as well as oligo-HA were entrapped in macroporous Ch-based matrices where Ch was previously cross-linked with GA or Gen. Two different methods were used; in particular, Method 1 was carried out via HA entrapment within the hydrogel volume, whereas Method 2 provided HA distribution and adsorption onto the hydrogel surface. The properties of the matrices, in particular their swelling behavior and degradation, were shown to be a function of the technique used and of the molecular weight of the entrapped HA. Moreover, for the first time, it was demonstrated that cell adhesion and morphology as well as cell distribution and proliferation within the hydrogel matrix were dependent upon the technique used for the preparation of the composite Ch/HA matrices.

The objectives of the study were as follows: (1) to develop two methods for preparing macroporous composite Ch/HA hydrogels based on covalently cross-linked Ch and low molecular weight HA; (2) to investigate some properties (such as swelling and in vitro degradation) and structures of the hydrogels; (3) to evaluate the hydrogels in vitro as potential biodegradable matrices for tissue engineering.

## 2. Materials and Methods

### 2.1. Chemicals

Chitosan (Mw 320 kDa, degree of deacetylation 88.5%) was purchased from Bioprogress (Shchelkovo, Russia), hyaluronic acid sodium salt (Mw 5 and 30 kDa) was from Shiseido (Tokyo, Japan) and glutaraldehyde was from Merck (Darmstadt, Germany). Genipin, 4′,6-diamidino-2-phenylindole dihydrochloride (DAPI) and lysozyme from chicken egg white (lyophilized powder, 100,000 U/mg) were purchased from Sigma (St. Louis, MO, USA). Dulbecco’s modified Eagle’s medium (DMEM), glutamine, sodium pyruvate, streptomycin, penicillin, phosphate buffered solution (PBS, pH 7.4) and 3-(4,5-dimethylthiazol-2-yl)-2,5-diphenyl tetrazolium bromide (MTT) were from PanEco (Moscow, Russia). Fetal bovine serum (FBS) was purchased from PAA Laboratories GmbH (Pasching, Austria), 2-mercaptoethanol was from Loba Feinchemie (Fischamend, Austria), dimethyl sulfoxide (DMSO) was from Helicon (Moscow, Russia) and Calcein AM was from eBioscience (San Diego, CA, USA).

### 2.2. Preparation of the Macroporous Composite Chitosan/Hyaluronic Acid Hydrogels

In this study, two methods for HA entrapment into the Ch hydrogel were used, namely before (Method 1) and after (Method 2) cross-linking chitosan with genipin or glutaraldehyde. Method 1 was used to provide bulk modification of the Ch hydrogel, and Method 2 allowed us to get the surface modification of the hydrogel.

#### 2.2.1. Bulk Modification (Method 1)

First, the HA solution (2.3% *w*/*v*, 6.6 mL) and then the Gen (0.95% *w*/*v*, 0.9 mL) or GA (0.4275% *w*/*v*, 0.9 mL) solution were added dropwise to the Ch solution (2.5% *w*/*v*, 30 mL) and stirred (1000 rpm). The obtained mixture was incubated by stirring it (1200 rpm) for 1.5 h at room temperature, and it was then lyophilized using Alpha 1-4/2-4 (Christ, Hagen, Germany).

#### 2.2.2. Surface Modification (Method 2)

A Gen (0.12% *w*/*v*, 0.9 mL) or GA solution (0.0525% *w*/*v*, 0.9 mL) was added dropwise to a Ch solution (2.5% *w*/*v*, 30 mL) and stirred (1000 rpm), and the obtained solution was incubated at room temperature for 2 h and then frozen and freeze-dried. The obtained macroporous cross-linked hydrogel samples were incubated in a 2% (*w*/*v*) HA solution for 2 h. Then, they were washed twice with PBS (pH 7.4) and lyophilized again.

### 2.3. Characterization of Macroporous Covalently Cross-Linked Chit/HA Hydrogels

#### 2.3.1. Fourier Transform Infrared Spectroscopy

FTIR-spectroscopy of the initial polysaccharides and the fabricated hydrogels was realized with the use of a Spectrum Two FT-IR Spectrometer (PerkinElmer, Waltham, MA, USA) as described previously [26]. All spectra were initially collected in attenuated total reflectance mode and converted into transmittance mode. The spectra were normalized using the intensity of C–O stretching vibrations of a pyranose cycle band (1081 cm^−1^) as the internal standard.

#### 2.3.2. Confocal Laser Scanning Microscopy

The structures of the swollen hydrogel samples were analyzed via confocal laser scanning microscopy using a Nikon TE-2000 inverted microscope equipped with an EZ-C1 confocal laser (Nikon, Tokyo, Japan). The hydrogel samples were stained with Fluorescamine (0.3 μg/mL in acetone) to provide amino-specific staining. The excitation wavelength was 408 nm, and fluorescence signals were collected at 515 ± 30 nm. Image analysis software (ImageJ, National Institutes of Health, Bethesda, Maryland, USA) was used for 3D reconstruction of the hydrogel structure. To study the morphology of the obtained macroporous hydrogels, a quantitative evaluation of micrographs was carried out by calculation of an effective pore diameter (*d*) using Equation (1):

(1) d=L×S12

where *L* is a pore long axis length and *S* is a pore short axis length. The mean pore size was determined by randomly measuring at least 100 pores for each hydrogel sample.

#### 2.3.3. Hydrogel Equilibrium Swelling Degree Measurements

The swelling degree of the obtained hydrogels was studied using a gravimetric method. For this purpose, the samples (5 × 5 × 2 mm) were incubated in DMEM at 37 °C for 24 h. The weight of the swollen hydrogel was determined to be the difference between the hydrogel weight and liquid weight on the balance plate after hydrogel removal. The swelling ratio (*Sw*) of the hydrogels was calculated using Equation (2):

(2) $$Sw(\frac{ml}{g})=\frac{M_{w}-M_{d}}{M_{d}}\times \rho$$

where *ρ* is the density of the solution, *Mw* is the weight of the sample after immersion in the medium and *Md* is the weight of the dried sample.

#### 2.3.4. Study of Enzymatic Degradation of the Hydrogels In Vitro

The degradation of the hydrogel samples was carried out in PBS (pH 7.4) containing 2 mg/mL lysozyme at 37 °C for 7, 14 and 21 days. The samples in PBS (pH 7.4) without lysozyme were used as controls. After 7, 14 and 21 days, the hydrogels were removed from the solution, washed with milli-Q, dried at 50 °C to constant weight and weighed. The weight loss (*Wl*) was calculated using Equation (3).

(3) $$Wl(\%)=\frac{M_{i}-M_{t}}{M_{i}}\times 100$$

where $M_{i}$ is an initial weight of the hydrogel sample and $M_{t}$ is the weight of the dried hydrogel sample.

### 2.4. Cell Cultivation in the Hydrogels

In the current study, mouse fibroblasts (L929) from the Collection of Vertebrate Cell Cultures (Institute of Cytology, Russian Academy of Sciences) were used. The L929 cells were cultured in DMEM supplemented with 10% FBS and containing 2 mM L-glutamine, 1 mM sodium pyruvate, 50 μM 2-mercaptoethanol, 100 μg/mL streptomycin and 100 U/mL penicillin. The cells were cultured in a 5% CO_2_ humidified atmosphere at 37 °C (CO_2_ incubator Heraeus B5060 EK/CO_2_, Hanau, Germany).

#### 2.4.1. Hydrogel Sterilization

The hydrogel samples were sterilized via incubation with 70% ethanol for 1 h. After sterilization, the samples were washed 3 times with PBS (pH 7.4).

#### 2.4.2. In Vitro Cytotoxicity Study

The cytotoxicity of the hydrogel samples was studied via an extract test using L929 fibroblasts as model cells. For this purpose, the previously sterilized hydrogel samples were incubated with the culture medium (25 mg per 1 mL of medium) at 37 °C, and supernatants (extracts) were collected after 24 h. Then, the cells were added to a 96-well plate (10^4^ cells per well) and incubated in a CO_2_ incubator (37 °C, 5% CO_2_). The medium in each well was replaced with 100 μL of the extracts after 24 h of incubation. The cells cultivated in the medium without the extracts were used as a control. Cell viability was determined via MTT assay. For this purpose, the extracts were replaced with 100 μL of a MTT solution (0.5 mg/mL DMEM) and then incubated at 37 °C for 1 h. Formazan crystals formed in the living cells were dissolved after adding DMSO (100 μL per each well), and optical density was measured at 540/690 nm using a Titertek Multiskan MCC/340 plate reader (Flow Laboratories, McLean, VA, USA). Relative cell viability (*V*) was calculated according to Equation (4):

(4) $$V(\%)=({OD}_{t}/{OD}_{c})\times 100$$

where ${OD}_{t}$ is the optical density in testing wells and ${OD}_{c}$ is the optical density in the control wells. Results are expressed as the mean ± standard deviation for three replicates.

#### 2.4.3. Study of Cell Proliferation in the Hydrogels

Before cell seeding, the sterile hydrogel samples were previously incubated in the culture medium at 37 °C for 24 h. Then, cells were seeded by dropping cell suspension directly onto the hydrogel samples (2 × 10^4^ cells/sample). Cell viability was evaluated with an MTT assay after 7 days. For this purpose, the hydrogel samples with the cells were transferred to a fresh 96-well plate, 100 μL of the MTT solution in DMEM (0.5 mg/mL) was added to each well, and the plate was then incubated at 37 °C for 2 h. Then, formazan crystals were dissolved after adding DMSO (200 μL per well) to each well, and 100 μL aliquots were taken to measure optical density at 540/690 nm. In this study, the chitosan hydrogel samples cross-linked either with Gen or GA were used as negative controls, whereas the cell monolayer culture was taken as a positive control.

In order to take into consideration the impact of each hydrogel sample on the results of the MTT assay, an additional experiment was carried out. For this purpose, culture medium with FBS was added to the previously sterilized blank hydrogel samples (without cells), and the samples were placed in a CO_2_ incubator for 7 days. Then, cell suspensions were added into the 96-well plate (cell numbers ranging 5–20 × 10^4^ cells/well), and the plate was transferred to the CO_2_ incubator for 3 h. Finally, the pre-incubated hydrogel samples were added to the previously attached cells, and the MTT assay was carried out for both the cells cultivated in the presence of the hydrogel samples and the cells without them. For each sample, a calibration curve was plotted that shows the optical density for the cells cultivated in the presence of each hydrogel sample (abscissa X) versus the optical density for the cells without the hydrogel sample (ordinate Y). Based on the obtained curve, the optical densities were determined for all hydrogel samples.

The relative cell viability (V) for each sample was calculated according to Equation (4). Results are expressed as the mean ± standard deviation for three replicates.

#### 2.4.4. Study of Cell Morphology

The hydrogel samples for confocal microscopy were prepared as described previously (see Section 2.4.3.). After 7 days of cell cultivation, the cells were stained with Calcein AM vital dye and DNA fluorescent dye DAPI. For this purpose, a mixture of Calcein AM (5 μg/mL) and DAPI (10 μg/mL) in DMEM was added to the hydrogel samples, and the samples were incubated at 37 °C for 30 min. Then, the supernatants were replaced with the fresh culture medium, and the samples were observed using a confocal laser microscope (Nikon TE-2000, Tokyo, Japan). The excitation wavelengths were 360 nm for DAPI and 488 nm for Calcein AM, and fluorescence signals were collected in the range of 380–460 nm for DAPI and 500–530 nm for Calcein AM.

#### 2.4.5. Statistics

The data were analyzed using GraphPad Prism 5.0 software (Graph-Pad Software, San Diego, CA, USA). All values are expressed as mean ± standard error of at least three parallel replicates, and they were compared using one-way analysis of variance (ANOVA) with Dunnett’s Multiple Comparison Test as a post hoc test. Values of *p* ˂ 0.05 are considered significant.

## 3. Results and Discussion

In the current study, the hydrogels based on Ch cross-linked with Gen or GA and modified with low molecular weight HA (MW 30 kDa) or oligo-HA (MW 5 kDa) were obtained and characterized in terms of their structures, biocompatibility and their ability to support cell growth and proliferation.

### 3.1. Preparation of the Macroporous Ch/HA Hydrogels

The macroporous matrices were prepared via the lyophilization of hydrogels from Ch, which was cross-linked with Gen or GA. As is well-known, this approach allows one to obtain non-soluble Ch hydrogels, which demonstrate rather high swelling behavior. The conditions for preparation of the Ch/HA samples were chosen based on polyelectrolyte complex formation mechanisms described earlier [27], whereas chitosan gelation from Ch solution using Gen or GA was also reported by us previously [28]. The conditions for cross-linking Ch hydrogels, namely pH and Gen/NH_2_ ratio, were selected based on our previous results, in particular the dependence curves of gelation time on Gen concentration [29]. The results of the change in the elasticity modulus of the chitosan hydrogels are shown in the Supplementary Materials (Figure S1). As a result of chitosan cross-linking with GA, more rigid hydrogels were formed than those in the case of Gen. Thus, equilibrium values of the modulus of elasticity measured with single-wall compression were found to be twice as high for the chitosan hydrogels cross-linked with GA than for Gen cross-linked hydrogels, even with lower cross-linker content in the case of GA. We also took into account that the polymer system should be liquid for at least 1.5 h, which is needed for the degassing and casting of the polymer solution in special forms for freezing.

**Figure 1:**
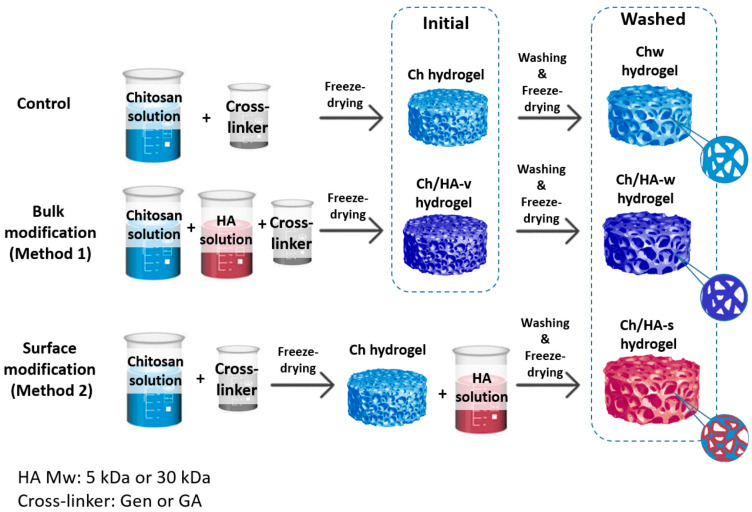
The scheme for the preparation of macroporous chitosan/hyaluronic acid hydrogels cross-linked with genipin (Gen) or glutaraldehyde (GA).

In the current study, two approaches to the preparation of the macroporous Ch/HA hydrogels with cross-linked Ch were developed (Figure 1). These approaches differ by way of the HA entrapment and its distribution in/on the Ch hydrogel.

In Method 1, the cross-linker was added to the mixtures of the Ch and HA solutions. As a result, one could suggest that HA molecules were distributed more or less evenly within the Ch hydrogel and formed polyelectrolyte complexes with Ch macromolecules (bulk modification). In Method 2, the chitosan macromolecules were first cross-linked with Gen or GA to get Ch cross-linked hydrogels, and after that, the HA solution was added, providing polyelectrolyte Ch/HA complex formation mostly on the surface of the Ch hydrogel. Thus, we obtained composite hydrogels, which differed in their structure due to HA macromolecule being distributed either mostly within the hydrogel volume (see Method 1) or over the hydrogel surface (see Method 2).

The hydrogel samples modified with HA over the surface (surface modification), hereafter referred to as Ch/HA-5s and Ch/HA-30s, differed only by HA molecular weight (5 and 30 kDa, relatively). These samples were additionally washed after the modification step and then freeze-dried. It should be noted that when using the freeze-drying technique, a number of freeze-drying cycles could affect the hydrogel structure. In order to take this effect into account, an additional set of the bulk-modified hydrogels was prepared and evaluated in the current study. For this purpose, the bulk-modified Ch/HA-5v and Ch/HA-30v hydrogels as well as the Ch hydrogels without HA (a control) were also washed with PBS (pH 7.4) and then lyophilized.

Thus, as seen in Table 1, two sets of the samples were prepared:

(1) Initial samples. These samples were obtained using Method 1 (bulk modification) for Ch/HA-5v, Ch/HA-30v and the non-modified Ch hydrogels in which Ch was cross-linked with Gen or GA.

(2) Washed samples. This set of samples can be divided in two parts, the first part being a subset of samples from the initial samples (1) but additionally washed with PBS (pH 7.4) after preparation and lyophilized (see Chw, Ch/HA-5w and Ch/HA-30w). The second subset of samples was prepared using Method 2 (surface modification) (see Ch/HA-5s and Ch/HA-30s) in which Ch was first cross-linked with Gen or GA, and then the hydrogels were incubated in the HA solution and finally washed with PBS (pH 7.4).

**Table 1:** A list of the prepared hydrogel samples and conditions for their preparation.

Sets	Samples	Ch Concentration, %	pH	HA Mw, kDa	Washing Conditions	Ch/HA, *w*/*w*	[Gen/GA]/[NH2] Ratio mol/mol	Gen Concentration, %	GA Concentration, %
Initial	Ch	2.5	5.6	-	-	-	0.01	0.12	0.0525
	Ch/HA-5v	2.5	5.6	5	-	5:1	0.01	0.95	0.4275
	Ch/HA-30v	2.5	5.6	30	-	5:1	0.01	0.95	0.4275
Washed	Chw	2.5	5.6	-	PBS (7.4)	-	0.01	0.12	0.0525
	Ch/HA-5w	2.5	5.6	5	PBS (7.4)	5:1	0.01	0.95	0.4275
	Ch/HA-30w	2.5	5.6	30	PBS (7.4)	5:1	0.01	0.95	0.4275
	Ch/HA-5s	2.5	5.6	5	PBS (7.4)	Surface mod	0.01	0.12	0.0525
	Ch/HA-30s	2.5	5.6	30	PBS (7.4)	Surface mod	0.01	0.12	0.0525

### 3.2. Characterization of the Hydrogels

**Figure 2:**
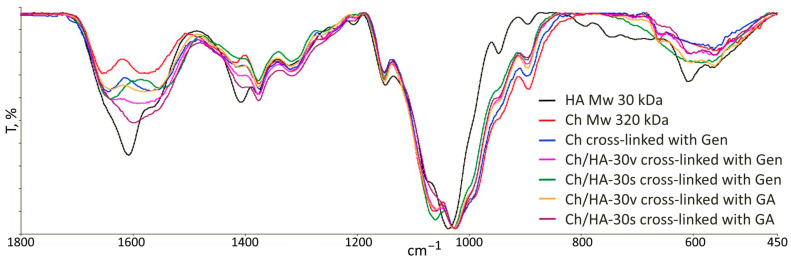
FTIR spectra of the initial polysaccharides and macroporous chitosan/hyaluronic acid hydrogels with cross-linked chitosan.

The FTIR spectra of the initial polysaccharides and the fabricated hydrogels are presented in Figure 2. The spectra of chitosan and hyaluronan show all well-resolved characteristic bands. The intense group of bands that extends from 1500 to 1700 cm^−1^ appears for all hydrogel samples. This group is the superposition of amide I and II bands and C=O and COO- bands. The main changes, which could be expected due to chitosan crosslinking and Ch/HA polyelectrolyte complex formation, overlap with the carboxylate ion stretching vibrations (about 1580 cm^−1^).

#### 3.2.1. Study of the Hydrogel Structures

To provide rather large specific surfaces for cell attachment and growth, hydrogels should have macroporous structures with open, interconnected geometry [30]. An interconnected, porous structure with pores of optimal size is known to stimulate cell growth, provide uniform cell distribution and spreading and promote neovascularization. In addition, these parameters are crucial in terms of effective mass and gas exchanges, which allows cells to be supplied with nutrients and oxygen [31].

In this study, the macroporous structures of the hydrogels were obtained via freeze-drying. As is well-known, the properties of the system to be frozen have a great influence on the formation of the macroporous structures. Varying the composition of a polymer system for hydrogel preparation allows for the formation of matrices that can differ in structure (morphology, average pore size, pore size distribution, etc.). As a result, the obtained structures can affect cell localization and distribution within the hydrogels.

**Figure 3:**
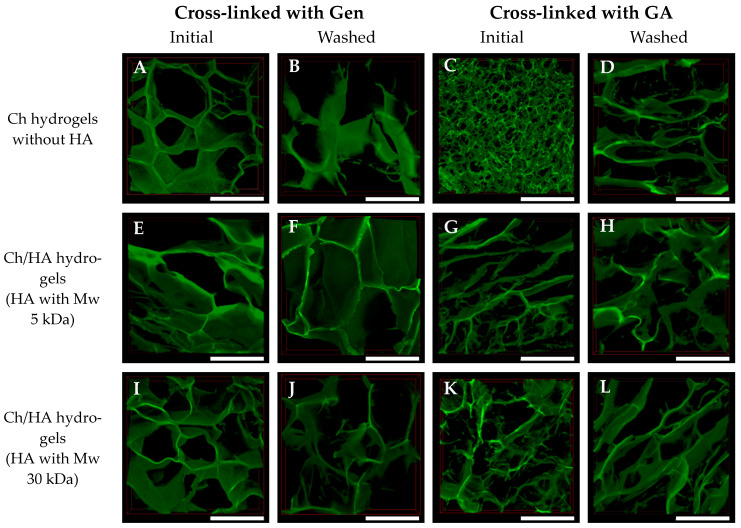
3D reconstructions of the swollen macroporous chitosan (**A**–**D**) and composite chitosan/hyaluronic acid hydrogels in which Ch was cross-linked with genipin (Gen) (**A**,**B**,**E**,**F**,**I**,**J**) or glutaraldehyde (GA) (**C**,**D**,**G**,**H**,**K**,**L**). Bulk modification (Method 1) with HA Mw 5 kDa (**E**–**H**) or 30 kDa (**I**–**L**). Scale bar is 500 µm.

**Figure 4:**
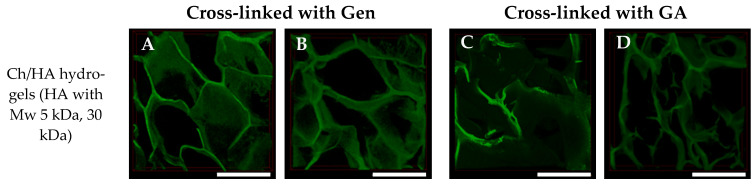
3D reconstructions of the swollen macroporous chitosan/hyaluronic acid hydrogels in which Ch was cross-linked with genipin (Gen) (**A**,**B**) or glutaraldehyde (GA) (**C**,**D**). Surface modification (Method 2) with HA Mw 5 kDa (**A**,**C**) or 30 kDa (**B**,**D**). Scale bar is 500 µm.

The structures of the swollen hydrogels were studied using CLSM. 3D reconstructions of these hydrogel samples are shown in Figure 3 and Figure 4. Some differences in the swollen hydrogels’ structures as function of their composition, type of cross-linking agent and preparation method were observed.

**Figure 5:**
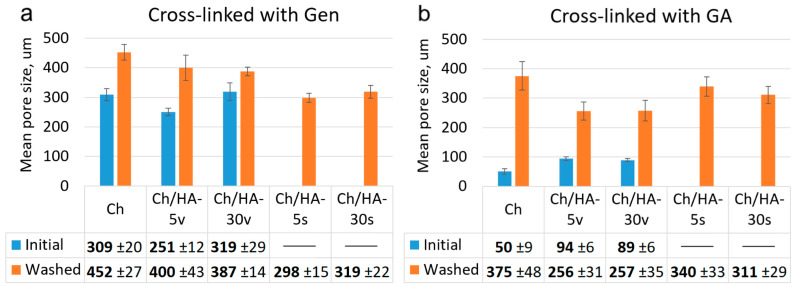
Mean pore sizes of the chitosan (Ch) and chitosan/hyaluronic (Ch/HA) hydrogels in which Ch was cross-linked with genipin (**a**) or glutaraldehyde (**b**). The Ch/HA-v hydrogels were prepared via bulk modification (Method 1), and the Ch/HA-s hydrogels were prepared via surface modification (Method 2).

The mean pore sizes for all hydrogel samples are shown in Figure 5. The pore sizes of the GA cross-linked hydrogels were smaller than those of the samples cross-linked with Gen, as GA is a more reactive cross-linking agent [32]. Therefore, in the case of GA, a formation of smaller ice crystals at freezing occurred, and as a result, an arrangement of denser structures was observed. For the most compact Ch hydrogel cross-linked with GA, an average pore diameter was 50 ± 9 μm. After an additional freezing cycle and washing of the sample, the pore size increased up to 375 ± 48 μm. The pore size increased because of the repeated swelling and subsequent freezing, and novel pores formed due to a partial destruction of the hydrogel structure as a result of growing ice crystals.

An entrapment of HA macromolecules into the composite hydrogel in which Ch was cross-linked with GA led to an increase in average pore size. A formation of rather big pores simultaneously with small pores was observed in the case of initial Ch/HA hydrogels prepared via bulk modification (Method 1). Thus, the mean pore size of 50 μm for the Ch hydrogel increased up to 94 ± 6 μm for the Ch/HA-5v sample. Moreover, an additional freezing cycle of these hydrogels resulted in an enhancement of the pore size up to 256 ± 31 μm for Ch/HA-5w sample. The pore sizes of the hydrogels prepared via surface modification (Method 2) were 340 ± 33 μm and 311 ± 29 μm for the Ch/HA-5s and Ch/HA-30s samples, respectively, in which Ch was cross-linked with GA, whereas in the case of the Ch/HA samples based on Ch cross-linked with Gen, the average sizes were 298 ± 15 µm (for Ch/HA-5s) and 319 ± 22 µm (for Ch/HA-30s). This could be explained by the washing of the samples after surface modification.

In the case of cross-linking with Gen, both Ch and Ch/HA hydrogels prepared via bulk modification were found to have higher mean pore sizes (within a range of 230–320 μm) than those in the hydrogels in which Ch was cross-linked with GA (94 ± 6 and 89 ± 6 μm for Ch/HA-5v and Ch/HA-30v samples, respectively). One can also see that additional washing resulted in the increase of the mean pore sizes of the non-modified and bulk-modified hydrogels cross-linked with Gen up to 387 ± 14 μm (for the Ch/HA-30w sample) and 400 ± 43 μm (for the Ch/HA-5w sample). The biggest pores (452 ± 27 μm) were obtained for the Chw hydrogel in which Ch was cross-linked with Gen after additional hydrogel washing followed by the freeze-drying step.

Pore size is one of key parameters for cell cultivation within matrices. On one hand, this is the parameter that depends upon the composition of the hydrogel used. On the other hand, different kind of cells could prefer matrices that differ in mean pore sizes. To provide diffusion of nutrients and metabolites at cell cultivation, the matrices with an average pore size of >50 μm are desirable [33]. Thus, we could suggest that our hydrogels with the mean pore sizes mentioned previously were suitable to support cell growth and proliferation. However, it should also be mentioned that vascularization within the hydrogel is also dependent upon its pore size. For instance, the depth and rate of vessel formation were higher for matrices with mean pore sizes of 50–150 μm than those for hydrogels with smaller pores within a range of 25–70 μm [34,35]. As for the matrices with pores >200 µm, the development of bigger vessels was revealed, which was not the case for the matrices with smaller pores [36]. Cell proliferation and/or differentiation are known to depend upon the porosity of the hydrogel, in particular mean pore sizes. Thus, matrices with pores within a range of 70–120 µm, in contrast to those with pores ranging from 10 to 70 µm, were shown to better support chondrocyte proliferation as well as accumulation of type II collagen and glycosaminoglycans [35]. As for the differentiation of mesenchymal stromal cells into chondrocytes and the repair of cartilage defects, they were more effective in poly(ε-caprolactone) matrices with pores of 400 µm than in those with pores of 100–200 µm [37]. It has also been reported that matrices with a pore size within a range of 380–405 µm demonstrated chondrocyte growth, whereas matrices with pore sizes from 186 to 200 µm promoted fibroblast proliferation [38].

Thus, in our study, hydrogels with pore sizes in the range of 50–450 μm were prepared. Therefore, we expected that the matrices with these pore sizes were suitable for the cultivation of cells.

#### 3.2.2. Study of the Hydrogel Swelling

As is well-known, the swelling behavior of the hydrogels is of great importance, as it allows one to estimate cells’ ability to survive within the matrix. In addition, swelling properties could affect degradation rate. Earlier, the degradation rate was shown to increase along with swelling degree enhancement [39,40]. Moreover, the mechanical properties of wet hydrogels were found to be significantly reduced [9,22], which could negatively affect cell adhesion, morphology and proliferation. Regulation of the hydrophilic–hydrophobic balance of the hydrogels is of great importance in order to promote cell adhesion [41]. Because hyaluronic acid is hydrophilic, its entrapment could result in changing the swelling properties of the Ch hydrogels after modification with HA. Moreover, it was shown that introduction of high molecular weight HA into Ch hydrogels resulted in an enhancement of pore size, swelling ratio and degradation rate [22].

**Figure 6:**
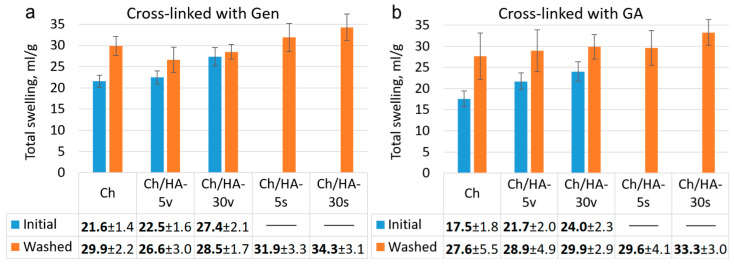

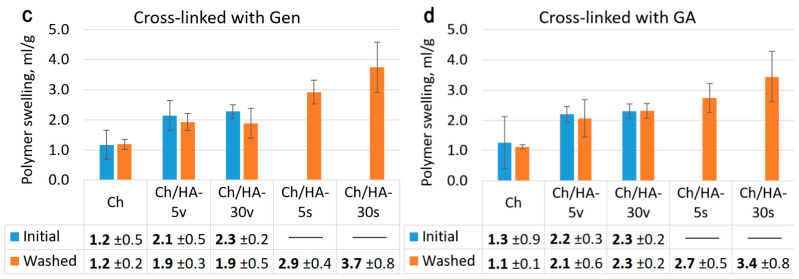
Total (**a**,**b**) and polymeric (**c**,**d**) swelling degrees of the hydrogels based on Ch cross-linked with genipin (**a**,**c**) or glutaraldehyde (**b**,**d**) after incubation of the hydrogels in culture medium (DMEM) for 24 h.

The total swelling of the hydrogel samples is considered to be a sum of two parameters, namely polymer swelling, which is related to the swelling capacity of the hydrogel walls, and structural swelling, which characterizes the amount of water retained in the pores. As for our study, the hydrogels’ swelling capacity measurements are shown in Figure 6. It can be seen that the modification of the chitosan hydrogels with HA as well as additional washing and freeze-drying markedly affected the equilibrium swelling degree of the samples (Figure 6a,b). Minimal swelling degrees of 21.6 ± 1.4 and 17.5 ± 1.8 mL/g were found for the initial non-modified Ch hydrogels cross-linked with Gen and GA, respectively. The bulk modification with HA led to these increased values (27.4 ± 2.1 and 24 ±2.3 mL/g for Ch/HA-30v samples in which Ch was cross-linked with Gen or GA, respectively). For the hydrogels modified with oligo-HA (Mw 5 kDa), the swelling degree values either did not change or increased a little bit (see Figure 6a,b). However, after the additional washing cycle, the swelling degree values of both non-modified Ch hydrogels and Ch/HA samples after bulk modification increased. Thus, for washed Ch hydrogels, the enhancement was up to 29.9 ± 2.2 and 27.6 ± 5.5 mL/g for Ch hydrogels cross-linked with Gen or GA, respectively.

In the case of surface modification with HA (Method 2), swelling increased compared to the swelling values obtained for both the initial samples and the washed hydrogels after bulk modification (Method 1). Moreover, modification with HA (Mw 30 kDa) led to a significant increase in total swelling. The maximum equilibrium swelling degree values were 34.3 ± 3.1 mL/g and 33.3 ± 3.0 mL/g for two Ch/HA-30s samples in which Ch was cross-linked with Gen or GA, respectively. This increase could be explained by the impact of pore walls swelling on polymer swelling (see Figure 6c,d). This can be attributed to partial damage to Ch/HA polyelectrolyte complexes as a result of interaction with various ions in the cultivation medium (DMEM). The entrapment of oligo-HA (Mw 5 kDa) into the hydrogel composition led to a less pronounced increase in the swelling degree of the Ch/HA samples.

It should be noted that additional washing and freeze-drying did not affect the polymer swelling behavior of both non-modified Ch and Ch/HA samples after bulk modification (see Figure 6c,d). An increased equilibrium swelling of these hydrogels after washing could be explained by changes in the hydrogel structures and a retention of water within the hydrogel pores due to enhanced porosity as a result of re-freezing (see Figure 4 and Figure 5). Thus, an increase of mean pore sizes and porosity in the samples in which Ch was cross-linked with Gen or GA because of repeated swelling was observed.

These results are consistent with those reported earlier [22]. Correia et al. showed that HA entrapment leads to an increase in the swelling degree of the Ch/HA hydrogels compared to the swelling degree of the Ch hydrogel.

#### 3.2.3. Study of Enzymatic Degradation of the Hydrogels

**Figure 7:**
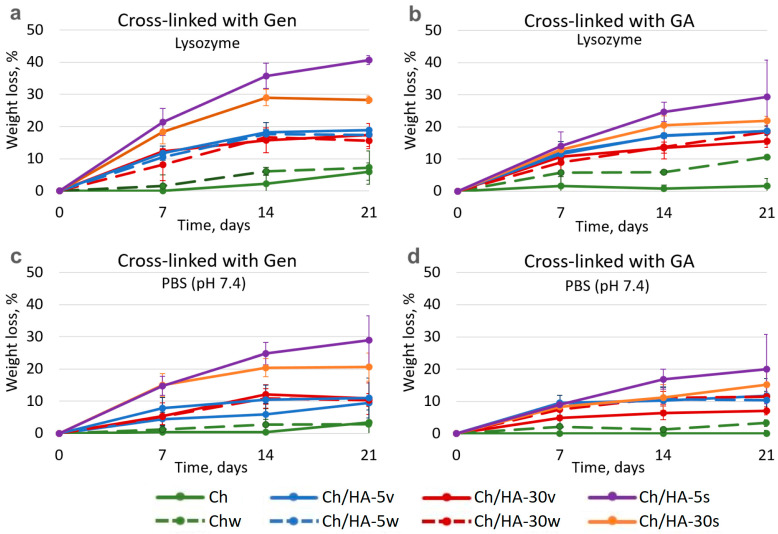
Weight loss of the hydrogels based on chitosan cross-linked with genipin (**a**,**c**) or glutaraldehyde (**b**,**d**) after 21 days of incubation in lysozyme solution (2 mg/mL, in PBS) (**a**,**b**) and PBS at pH 7.4 (**c**,**d**).

The study of matrix degradation behavior is of great importance, as it allows estimation of the time needed for growing cells to fill the pores (cavities) of the hydrogel and in parallel to synthesize an extracellular matrix, which should replace our polymer matrix. In addition, the degradation rate of the polymer matrix should be well-correlated with the rate of novel tissue formation. In order to provide an optimal tissue regeneration rate, the polymer matrix should decompose no faster than ECM is deposited. As is well-known, there are different mechanisms of hydrogel destruction, for instance, resorption and degradation under water and CO_2_ action, or degradation as a result of enzyme hydrolysis. Here, we studied biodegradation of Ch and Ch/HA hydrogel samples using a solution of lysozyme in PBS (pH 7.4) (Figure 7). Lysozyme is known to cleave chitosan macromolecules. Hydrogel degradation in PBS (pH 7.4) without lysozyme was used as a control. As seen in Figure 7, the degradation of all hydrogel samples in the lysozyme solution (in PBS) was faster than that in PBS (pH 7.4), whereas trends in the behavior of all the samples were preserved.

The composition of the hydrogels was found to influence hydrogel degradation behavior. The Ch/HA-5s and Ch/HA-30s samples were the weakest, whereas the non-modified Ch hydrogels cross-linked either with Gen or GA were the most stable. The Ch hydrogel samples cross-linked with GA were a bit more stable than those cross-linked with Gen. It can be assumed that that covalent cross-linking hampered the cleavage of Ch macromolecules via lysozymes due to steric hindrance. Moreover, hydrogel structure could also affect this process. For example, the Ch hydrogel cross-linked with GA had the densest structure, which could limit diffusion. Therefore, biodegradation proceeded more slowly, and weight loss was less than 2% after incubation for 21 days. Additional washing and lyophilization of Ch samples led to a slight enhancement of the weight loss rate. The most pronounced effect was found for GA-cross-linked Ch hydrogel, as the most drastic change in the hydrogel structure was observed for this sample (see Figure 4 and Figure 5).

As for the Ch/HA hydrogels, the entrapment of HA led to an increase in the weight loss of the samples. For hydrogels after bulk modification, HA molecular weight as well as washing did not markedly influence hydrogel degradation. Thus, the weight losses of these hydrogels were more or less similar; in particular, they were 15–19% after 21 days of incubation in the lysozyme solution.

As for the Ch/HA samples after surface modification, we observed faster weight losses than those for the Ch hydrogels. Moreover, the samples with oligo-HA (Mw 5 kDa) degraded faster than those with Mw of 30 kDa. Thus, the most pronounced effect (41%) was revealed for the Ch/HA-5s hydrogel sample in which Ch was cross-linked with Gen. As for the Ch/HA hydrogels in which Ch was cross-linked with GA, they degraded faster than the Ch samples as well, but they degraded more slowly compared to the Ch/HA hydrogels in which Ch was cross-linked with Gen. For instance, the weight loss of the Ch/HA-5s hydrogel with GA was 29% after 21 days of incubation in the lysozyme solution.

### 3.3. In Vitro Study

#### 3.3.1. Cytotoxicity Study of the Hydrogels

Because GA is rather toxic [42], there is increasing interest in using genipin as a cross-linker, which would impart stability and rigidity to biocompatible hydrogels. Genipin is 5–10 thousandfold less cytotoxic than glutaraldehyde [43]. The limiting factor for genipin widespread use is its rather high cost. Recently, a new method for genipin preparation from geniposide using *Fusarium solani* was reported [10]. In this context, Gen is a promising alternative to GA to improve the mechanical properties of Ch-based matrices [44]. In this study, we used Gen along with GA to prepare cross-linked hydrogels. Therefore, it was of great importance to evaluate the possible cytotoxic effects of both of these compounds.

**Figure 8:**
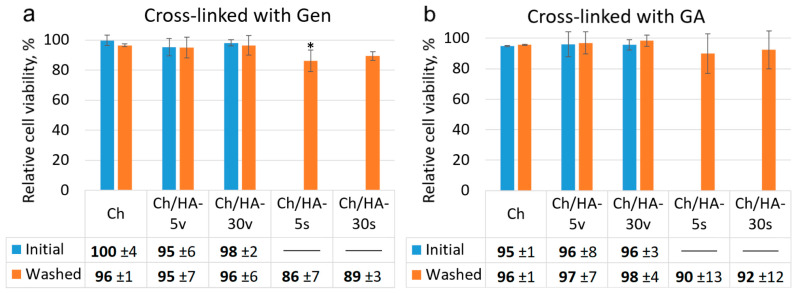
Viability of L929 mouse fibroblasts after 24 h incubation with the extracts of the hydrogels in which Ch was cross-linked with genipin (**a**) and glutaraldehyde (**b**). Results of MTT assay. Monolayer cell culture was taken as a control (100%). Data are expressed as the mean ± SD. Asterisk indicates significant difference versus control (* *p* < 0.05). Three parallel replicates were carried out for each sample.

The cytotoxicity of the hydrogels was studied using an extract test (Figure 8). This technique allows estimation of the cytotoxic effects of the compounds released from the matrix after incubating the hydrogel samples in DMEM (10% FBS) for 24 h. Cell viability was measured via MTT assay after cell cultivation in these extracts for 24 h. As seen in Figure 8, there was a 90% decrease of cell viability for the extracts of the Ch/HA hydrogels prepared via surface modification. This could be attributed to an acidic environment as a result of the partial destruction of a Ch/HA polyelectrolyte complex. For other hydrogels, we did not observe any decrease in viable cell numbers after cell cultivation in these extracts for 24 h compared to the control (monolayer cell culture in DMEM + 10% FBS).

#### 3.3.2. Growth of Cells in the Hydrogel Samples In Vitro

Hyaluronic acid as one of the key components of the ECM provides many specific interactions with growth factors, adhesive proteins and receptors. Therefore, HA entrapment into the chitosan hydrogels could alter the bioactivity of these matrices. To estimate the effects of the hydrogel properties on cell behavior, particularly cell adhesion, spreading and proliferation, mouse fibroblasts L929 were cultivated in the hydrogels for 7 days. Cell morphology was observed via CLSM, and cell proliferation was evaluated by using the MTT assay.

#### 3.3.3. Morphology of Cells in the Hydrogels

**Figure 9:**
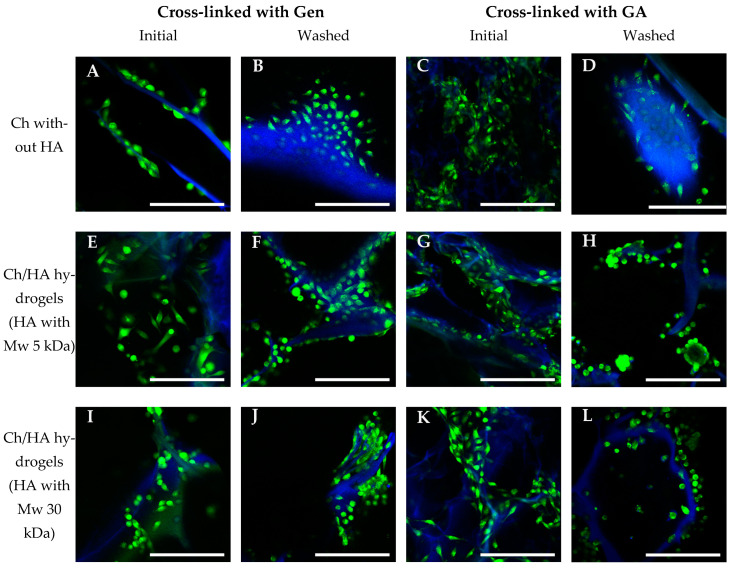
CLSM images of L929 mouse fibroblasts cultivated in chitosan (**A**–**D**)and chitosan/hyaluronic acid hydrogels in which Ch was cross-linked with genipin (Gen) (**A**,**B**,**E**,**F**,**I**,**J**) or glutaraldehyde (GA) (**C**,**D**,**G**,**H**,**K**,**L**) for 7 days. Bulk modification (Method 1) with HA Mw 5 kDa (**E–H**) or 30 kDa (**I**–**L**). Living cells were stained with Calcein AM (in green), and cell nuclei and hydrogel structures were stained with DAPI (in blue). Scale bar is 200 μm.

**Figure 10:**
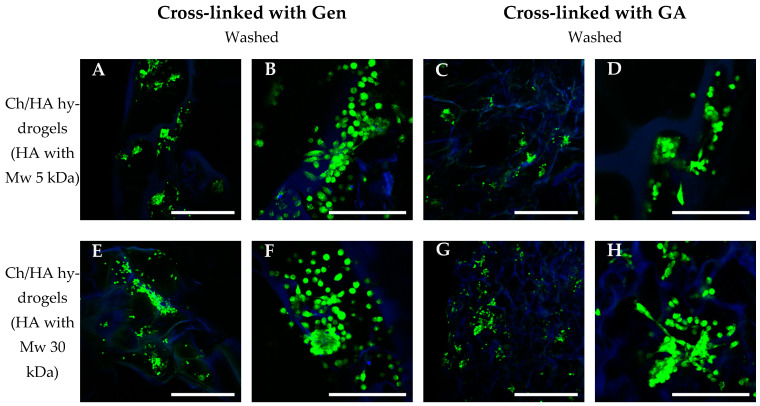
CLSM images of the L929 mouse fibroblasts cultivated in the chitosan/hyaluronic acid hydrogels in which Ch was cross-linked with genipin (Gen) (**A**,**B**,**E**,**F**) or glutaraldehyde (GA) (**C**,**D**,**G**,**H**) for 7 days. Surface modification (Method 2) with HA Mw 5 kDa (**A–D**) or 30 kDa (**E**–**H**). Living cells were stained with Calcein AM (in green), and cell nuclei and hydrogel structures were stained with DAPI (in blue). Scale bars are 500 (**A**,**C**,**E**,**G**) and 200 (**B**,**D**,**F**,**H**) μm.

As seen in Figure 9, the L929 cells were distributed evenly over the matrix surface in both cases of initial (non-modified) Ch hydrogels and Ch/HA samples after bulk modification. After 7 days of cultivation, the cells in these matrices were found to attach, spread well and form monolayers on the hydrogels’ surfaces. In contrast, in the Ch/HA hydrogels after surface modification, the cells were distributed less evenly, were not well-spread and formed multicellular aggregates (Figure 10). This could be explained by a negatively charged HA surface, which causes an electrostatic repulsion of negatively charged cell membranes [45]. As a result, the cells were spherical in shape and did not spread. As for the Ch hydrogels and the Ch/HA hydrogels (bulk modification) after washing, the cells in these samples were distributed evenly over the surface. However, the cellular aggregates in these hydrogels were also revealed (see Figure 9H,J).

Thus, it can be concluded that the surfaces of the Ch/HA hydrogels after bulk modification were better for L929 fibroblast adhesion, spreading and growth than those after surface modification.

#### 3.3.4. Cell Proliferation in the Hydrogels

**Figure 11:**
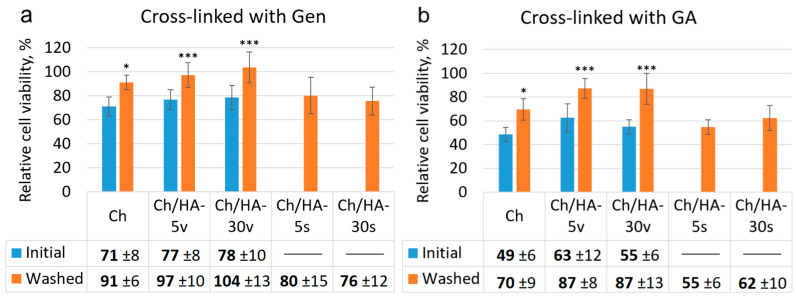
Viability of L929 mouse fibroblasts cultivated in the hydrogels in which Ch was cross-linked with genipin (**a**) and glutaraldehyde (**b**) for 7 days. Results of MTT assay. The monolayer cell culture (without the hydrogel sample) was taken as a control (100%). Data are expressed as the mean ± SD. Asterisk indicates significant difference versus control (initial Ch hydrogels) (*** *p* < 0.001; * *p* < 0.05). Three parallel replicates were carried out for each sample.

The growth and proliferation of cells within the hydrogel samples was studied via MTT assay (Figure 11). A number of viable L929 fibroblasts in the hydrogels were found to depend upon the hydrogel type. As can be seen in Figure 11, cell numbers for all hydrogels in which Ch was cross-linked with Gen were higher than those for all hydrogels in which Ch was cross-linked with GA. This fact can be explained by the smaller average pore sizes of the hydrogels from Ch cross-linked with GA (50–100 µm) compared to those of the samples in which Ch was cross-linked with Gen (>250 µm). The structures with smaller pores could have led to limited cell migration and reduced cell proliferation. It is worth noting that in the case of the washed samples, increased cell growth was revealed. After additional hydrogel washing and lyophilization, the numbers of viable cells were higher for the Ch hydrogels, and especially for those that were cross-linked with GA, than the same values for initial hydrogels. This could be also attributed to changes in the samples structure, as for the GA-cross-linked hydrogels, these changes were more pronounced. These results could be also explained by an enhancement of the specific surface of the samples due to their increased porosity. As a result, these changed hydrogel structures could contribute to the observed improved cell adhesion and growth. Thus, additional washing and lyophilization of the matrices affected the hydrogel structures by increasing their pore sizes and porosity, which in turn led to enhanced cell migration within the matrices. Therefore, the structures of the washed hydrogels were more favorable for cell growth and proliferation.

Modification of the Ch hydrogels by HA entrapment in both methods led to cell growth enhancement in the Ch/HA matrices. The bulk HA modification of the hydrogels led to increased numbers of viable cells. Moreover, between the initial bulk-modified samples and samples modified on the surface, their relative cell viability values were comparable. For instance, in the case of the hydrogels in which Ch was cross-linked with Gen, cell viability rates in Ch/HA-5v, Ch/HA-30v, Ch/HA-5s and Ch/HA-30s hydrogels were 77 ± 8%, 78 ± 10%, 80 ± 15% and 76 ± 12%, respectively. In the case of the hydrogels with GA, cell viability rates in Ch/HA-5v, Ch/HA-30v, Ch/HA-5s and Ch/HA-30s were 63 ± 12%, 55 ± 6%, 55 ± 6% and 62 ± 10%, respectively. Maximum cell viability values were found for the washed samples. Thus, as seen in Figure 11, the maximum number of living cells (104 ± 13%) was revealed for the washed Ch/HA-30w sample in which Ch was crossed-linked with Gen. It should be also noted that we did not find any significant differences in cell viability for the Ch/HA samples that differed in the molecular weight of hyaluronic acid used.

Thus, both methods for the modification of cross-linked Ch hydrogels via entrapment of hyaluronic acid allowed us to enhance cell growth and proliferation.

## 4. Conclusions

In this study, two different methods are proposed for the fabrication of cross-linked chitosan hydrogels modified via the entrapment of hyaluronic acid (Mw 5 kDa or 30 kDa) as a bioactive compound. In order to prepare the macroporous composite Ch/HA hydrogels based on polyelectrolyte complexes, hyaluronic acid was entrapped in the Ch hydrogels either via bulk modification (Method 1) or surface modification (Method 2). The chitosan macromolecules were cross-linked with GA or Gen.

All hydrogels were characterized in terms of their FTIR spectra, swelling behavior, structure, in vitro enzymatic degradation and their ability to support cell adhesion and growth. The effects of HA on the Ch/HA hydrogel properties mentioned previously were evaluated regarding the function of the method for HA entrapment, the molecular weight of the HA and the cross-linker (Gen or GA) used for Ch cross-linking. The swelling degree and degradation were found to depend on the method used and the composition of the Ch/HA hydrogel samples. Thus, HA entrapment into the Ch hydrogels led to an increase in the swelling degree as well as an enhancement of the degradation of the Ch/HA samples. Moreover, HA entrapment via surface modification (Method 2) resulted in bigger changes in these parameters than in the samples prepared using Method 1. All hydrogels were not toxic, which was confirmed in the extract test using the L929 mouse fibroblasts. The 3D cell growth and proliferation in the hydrogels were studied. Cell morphology and viability in the hydrogels were shown to depend on hydrogel composition and the preparation method used. The Ch/HA hydrogels after bulk modification promoted better cell adhesion and spreading as well as cell growth and proliferation compared to the samples prepared using Method 2 (surface modification). Moreover, additional washing and freeze-drying provided better cell adhesion and proliferation, whereas HA introduction into the hydrogels resulted in enhanced cell growth compared to the Ch samples.

Thus, by varying the Ch-based hydrogel composition and fabrication technique, macroporous composite Ch/HA hydrogels with highly porous interconnected structures were developed. A chitosan component of these hydrogels provided rather good cell adhesion, whereas a combination of Ch with HA enhanced cell growth and proliferation. The cross-linked chitosan hydrogels modified with hyaluronic acid could be promising for tissue engineering.

## Data Availability

Data is contained within the article.

## Supplementary Materials

The following supporting information can be downloaded at: https://www.mdpi.com/article/10.3390/polym15102371/s1, Figure S1: Kinetics of a change in the elasticity modulus (E) of chitosan (MM 320 kDa) hydrogels cross-linked with: (1) GA/NH2 (0.005 mol/mol); (2) Gen/NH2 (0.01 mol/mol). The value of the modulus of elasticity was determined using a modified Kargin balance using the method of single-axis compression under stepped loading. The samples had the form of tablets with a diameter of 20 mm and a height of 8 mm. The measurement accuracy was 0.01 mm and corresponded to a deformation of 0.125%.
