# Supplementary material for: Composite Hydrogels Based on Cross-Linked Chitosan and Low Molecular Weight Hyaluronic Acid for Tissue Engineering
Source: Polymers (Basel). 2023 May 19;15(10):2371. doi: 10.3390/polym15102371 (PMC10222357; doi:10.3390/polym15102371)
Supplement: Supplementary file 1 [file polymers-15-02371-s001.zip › polymers-2404334-supplementary.pdf]

Supporting Information:

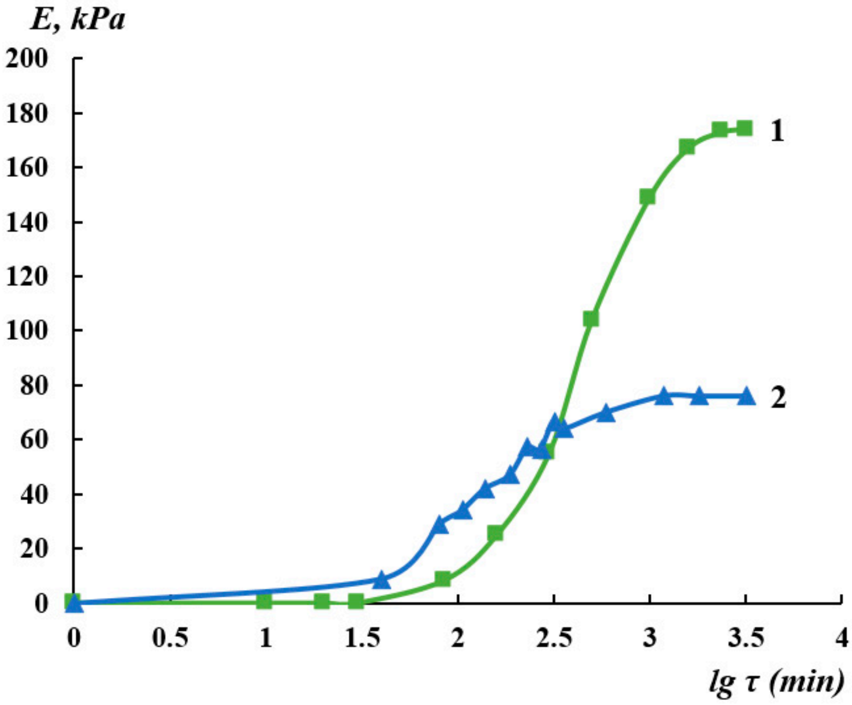

Figure S1. Kinetics of a change in the elasticity modulus (E) of chitosan (MM 320 kDa) hydrogels cross-linked with: 1) GA/NH2 (0.005 mol/mol); 2) Gen/NH2 (0.01 mol/mol). The value of the modulus of elasticity was determined using a modified Kargin balance using the method of single-axis compression under stepped loading. The samples had the form of tablets with a diameter of 20 mm and a height of 8 mm. The measurement accuracy was 0.01 mm and corresponded to a deformation of 0.125%.
